# Topologically nontrivial bismuth(111) thin films

**DOI:** 10.1038/srep21326

**Published:** 2016-02-18

**Authors:** Meng-Yu Yao, Fengfeng Zhu, C. Q. Han, D. D. Guan, Canhua Liu, Dong Qian, Jin-feng Jia

**Affiliations:** 1 Key Laboratory of Artificial Structures and Quantum Control (Ministry of Education), Department of Physics and Astronomy, Shanghai Jiao Tong University, Shanghai 200240, China; 2 Collaborative Innovation Center of Advanced Microstructures, Nanjing 210093, China

## Abstract

Using high-resolution angle-resolved photoemission spectroscopy (ARPES), the topological property of the three-dimensional Bi(111) films grown on the Bi_2_Te_3_(111) substrate were studied. Very different from the bulk Bi, we found another surface band near the 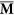 point besides the two well-known surface bands on the 30 nm films. With this new surface band, the bulk valence band and the bulk conduction band can be connected by the surface states in the Bi(111)/Bi_2_Te_3_ films. Our band mapping revealed odd number of Fermi crossings of the surface bands, which provided new experimental evidences that Bi(111)/Bi_2_Te_3_ films of a certain thickness can be topologically nontrivial in three dimension.

Topological insulators (TIs) possessing the topological surface state have been extensively studied in the last several years^1^,^2^,^3^,^4^,^5^,^6^,^7^,^8^,^9^,^10^. The first experimental confirmed three dimensional (3D) TI is Bi_1*−x*_Sb_*x*_ alloy^4^. Bismuth (Bi) itself is famous for the novel surface states^11^. Though its surface states are very robust^12^,^13^,^14^, bulk Bi is topologically trivial in theory^3^. However, the calculated and measured band structures do not agree well. We illustrate the bands of bulk Bi(111) near the Fermi level including surface states according to the local-density approximation (LDA) calculations^15^ in Fig. 1a. Two spin splitting surface bands (“S1” and “S2”) are shown in calculations. According to the Kramers theorem, S1 and S2 must be degenerate at the time-reversal invariant momenta (TRIM) points, 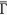 and 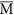 points in the surface hexagonal Brillouin zone. However, the experimental findings were very complicated. Figure 1b shows the sketch of reported ARPES bands of bulk Bi(111)^11^,^15^,^16^,^17^,^18^,^19^,^20^,^21^. At 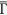 point, S1 and S2 are degenerate (merge into bulk valence band (BVB) together), but they are not degenerate at 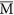 point. In order to satisfy Kramers theorem, S1 must has a partner (another surface band) at 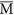 point or merge into bulk conduction band (BCB). Experimentally, the topological property of Bi will depend on the detailed band structure near the 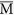 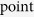. Unfortunately, no information about the partner and BCB near the 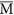 point has been obtained by ARPES. Because the discrepancy between calculations and experiments, recent work claimed that bulk Bi is topologically nontrivial^21^.

On the other hand, Bi(111) films are also very interesting. Recent transport measurements on Bi(111) thin films and Bi nanoribbons revealed some experimental evidences of the existence of the topological protected surface states^12^,^13^,^14^. New theory was developed to show that Bi(111) thin films could be a 3D TI like^13^. Other work also showed that the topological property of Bi film could be affected by the lattice constant^22^,^23^. Therefore, the topological property of Bi films may be different from bulk Bi. Many ARPES experiments have been carried out on Bi(111) films grown on the Si(111) substrate^24^,^25^,^26^,^27^,^28^. The reported ARPES spectra near 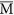 point on Bi(111) films are very similar to the bulk Bi^27^. Recently, single crystalline Bi(111) films were obtained on Bi_2_Te_3_(111) substrate^29^,^30^,^31^,^32^. This system provides us a new opportunity to explore the topological property of Bi(111) films. In this work, we studied the electronic structures of Bi(111)/Bi_2_Te_3_ films using high resolution ARPES. Interestingly, we found three surface bands near Fermi level on the 30 nm films. Our band mapping gives a better visualization that 30 nm Bi(111)/Bi_2_Te_3_ film is topologically nontrivial.

Figure 2a shows the RHEED pattern of 30 nm Bi(111) films. RHEED is sensitive to the surface layers. Sharp and line-like pattern indicates the high-crystalline-quality and flat surface. Steps of single biatomic-layer (BL) were clearly observed by scanning tunneling microscopy (STM) (Fig. 2b). The distance between two adjacent lines in RHEED pattern is inversely proportional to the in-plane lattice constant (*a*_*Bi*_) of Bi. In Fig. 2c, *a*_*Bi*_ increases from 4.38\pm0.02\AA (same as Bi_2_Te_3_ substrate) to 4.54\pm0.02\AA (bulk value of Bi) below about 13 nm and then keeps constant. It is worth noting that the most part of the crystal structure analysis of Bi(111) thin films has been already done^22^ and it is almost the same as that of the bulk single crystal. Only the change of the in-plane lattice constant in 0.1% order of magnitude is not clear due to our experimental uncertainty. Smooth change of *a*_*Bi*_ and lack of dislocations confirmed by STM indicate that strain in the film is very likely released through continuous increase of *a*_*Bi*_ in each BL.

Figure 3a,b present the ARPES spectra of 30 nm and 20 nm Bi(111)/ Bi_2_Te_3_ films near 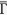 point along 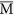
*-*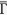-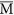 direction. Consistent with previously works, two surface states are observed (“S1” and “S2”). They merge at 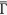 point and split when away from 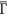 point. Centered around 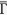 point, there is a hole band (green dotted line is a guide for the eyes) that is the BVB^27^. We found that the Fermi energy of two films is different. Figure 3c,d show the energy distribution curves (EDCs) at 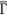 point compared with the EDC from Au for 30 nm and 20 nm films, respectively. Clearly, in Fig. 3c, there is an energy gap. Estimated from the leading edge of EDC, the energy gap is about 13.6 ± 2.5 meV. It should be noted that this experimental gap is not the exact gap of BVB because we only measure at a single k_z_ point (one k_z_ point corresponds to one incident photon energy). On those films, we observed clear BVB signals only under several incident photon energies, so we do not know the band maximum position of the BVB along k_z_. In Fig. 3d, no energy gap was observed on 20 nm films. Fermi level of 20 nm film is slightly lower than that in 30 nm film. Energy gap was also observed on ~ 70 nm Bi(111)/Si(111) films recently, which was attributed to quantum size effect^33^. Near the 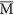 point, we find that the S1 band is very close to the Fermi level in 20 nm film, which is the same as the published ARPES spectra in bulk Bi(111)^11^,^15^,^16^,^17^,^18^,^19^,^20^,^21^. Interestingly, in 30 nm film, we find the S1 band is lower in energy (or Fermi level is higher), which let us observe new spectral features near the 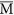 point. We will discuss it below.

Figure 4a–f show the ARPES spectra and corresponding EDCs of 30 nm Bi(111) films near the 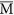 point along 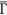-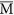-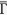 direction using different incident photon energies. The center of the spectra is at 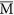 point (k = 0.8 \AA^*−*1^, Brillouin zone boundary). Shown in Fig. 4a, two surface states (S1 and S2) are observed (green dash lines are guides for the eyes). S2 is “W”-shape centered at 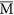 point. In Fig. 4a, S1 is easy to be traced from EDC peaks where k < 0.7 \AA^*−*1^ and become difficult to do so where 0.7 \AA^*−*1^ < k < 0.8 \AA^*−*1^. In this region, we have to follow the momentum distribution curves (MDCs) and secondary derivative image (SDI) plot that can enhance the dispersion relation to obtain the complete dispersion of S1 band. Figure 4g presents the MDCs near the Fermi level extracted from Fig. 4a. Green dotted lines mark the possible MDC peaks of S1 band, which implies a “W”-shape band. SDI plot in Fig. 4h near the 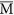 point also indicates S1 band has a “W”-shape. Due to the weak signals near 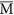 point, the extracted dispersion relation of S1 band has some uncertainty, but nevertheless we observed a “W”-shape S1 band.

Beside S1 and S2 bands, we observed another band (“S3”, blue dashed line in Fig. 4). The intensity of S3 band is not very strong, but we can trace its dispersion on EDCs. Figure 4i shows the high resolution EDCs near 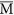 point (h*ν* = 30 eV). The momentum is from 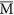 point towards 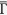 point (0.39 \AA^*−*1^ ≤ k ≤ 0.8 \AA^*−*1^). In Fig. 4i, two peaks from S1 and S3 bands are clearly resolved in most of EDCs (green and blue dotted lines mark the EDC peaks of S1 and S3 bands, respectively). S3 band is a shallow electron-like band that disperses from 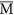 point at about 20 meV below Fermi level towards 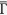 point. The exact Fermi crossing point of S3 band is affected by S1 band. We will give an estimate value below. Figure 4j shows the corresponding ARPES spectra of Fig. 4i. Four EDCs extracted from Fig. 4j are presented in Fig. 4k. The momentum positions of four EDCs are marked by the red lines in Fig. 4j. At k = 0.70 \AA^*−*1^ (EDC-4), S1 and S3 bands are well separated indicated by green and blue arrows. At k = 0.60 \AA^*−*1^ (EDC-3), S1 and S3 bands become closer but still resolvable. At k = 0.56 \AA^*−*1^ (EDC-2), single peak from S1 band was observed. At k = 0.43 \AA^*−*1^ (EDC-1), no peak is observed near Fermi level, which indicates that both S1 and S3 bands are already above Fermi level. Therefore, we estimate the crosses point of S3 band is roughly between 0.6 \AA^*−*1^ and 0.5 \AA^*−*1^.

Where does S3 band come from? There are three most likely possibilities. The first possibility is that S3 band is the missing BCB of Bi. However, S3 band is much flatter than BCB obtained in the calculations^15^. So we can exclude this possibility. The second possibility is that S3 band is an impurity band. Impurity band is non-dispersive and should be detected at all or most of the momentum positions. In contrast, S3 is dispersive and only detected within small region of momentum. Therefore, S3 cannot be due to impurity band. The third possibility is that S3 band is a surface state. To check this, we did the photon energy dependent ARPES measurements to change the k_z_. 80% of the momentum space from Brillouin zone center to Brillouin zone boundary along <111*>* direction are covered. The in-plane dispersion of S3 band barely changes in Fig. 4a–f. Figure 4l shows the EDCs at 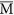 point as a function of the incident photon energy. Though the intensity varies, the peak position of S3 band does not change, which indicates that S3 band is very likely a surface state. Similar surface band was observed in 3D TI – Bi_1−x_Sb_x_^4^ crystals, while ARPES experiments on MBE grown Bi_1−x_Sb_x_ films did not find this additional surface band^34^. The additional surface band in Bi_1−x_Sb_x_ crystals may due to surface imperfection in the cleaved surface^34^. Our Bi films are grown by MBE method with high quality surface (Fig. 1b), so we think the S3 band is very unlikely due to surface imperfection.

According to the Kramers theorem, S1 must be degenerate with another surface band or merge into bulk band. In our films, BCB is still missing in ARPES spectra, however the third surface band S3 is observed. Our high resolution ARPES results suggest that S1 band is degenerate with S3 band at 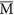 point. It is worth noting that S3 band was only observed on 30 ( ± 3) nm film. We do not know the exact mechanism. We guess that the special crystal structures of Bi/Bi_2_Te_3_ films may play an important role. Bi/Bi_2_Te_3_ films consist of 13 nm Bi layers with compressed lattice and additional Bi layers with uncompressed lattice. The ratio of the lattice-compressed and uncompressed layers varies for Bi films with different thickness. The electronic structure of Bi/Bi_2_Te_3_ films should be determined by the detailed combination of lattice-compressed and uncompressed Bi layers. Theoretical input will be very important to understand our experimental findings. However, as we discussed in the introduction, LDA calculations do not explain the experimental results very well even for bulk Bi. In addition, it is extremely difficult to do first principle calculations on the electronic structure of the Bi/Bi_2_Te_3_ films with non-uniform lattice constant. So far, the scientific and technical problems prevent us from comparing our experimental observation with theoretical calculations.

Figure 5 presents the ARPES spectra of 30 nm Bi(111)/Bi_2_Te_3_ films from one TRIM point (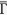) to another TRIM point (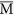). Green dashed lines mark the surface states. There are five Fermi crossing points indicated by the white arrows. Similar to Bi_1*−x*_Sb_*x*_^4^, odd number of Fermi crossing of surface states implies that 30 nm Bi(111)/Bi_2_Te_3_ is topologically nontrivial. Honestly, we don’t know why Bi(111)/Bi_2_Te_3_ is topological nontrivial. One possibility is lattice effect. Self-consistent GW calculations suggest that Bi can be a topological nontrivial if the in-plane lattice is compressed by only 0.4%^23^. The required change of in-plane lattice is very small. It is possible in the thin films. High resolution gracing XRD is needed to determine the accurate in-plane lattice constant (change in 0.4% ~ 0.018 \AA is smaller than our RHEED resolution) in the future to check the possibility. Another possibility is that bulk Bi is actually topological non-trivial. In previous ARPES studies on Bi(111), only S1 and S2 bands were observed near 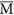 point. Based on the tight binding calculations that reproduced the bulk Fermi surface^21^,^22^,^35^, experimental S1 band merges with BCB^11^,^21^, suggesting Bi could be topological nontrivial. However, parity analysis based on the first principle calculations shows Bi is topological trivial^3^,^23^, which means S1 band should not merge with BCB. The very recently state of the art GW calculation^23^ shows that the bottom of BCB is the same as that in the tight binding calculations^35^, which again let the experimental S1 band merge with BCB. In our films (20 to 30 nm), it is clear that S1 and S2 bands do not merge at the 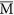 point, which suggests the non-trivial topology of the films considering the requirement of Kramers theorem. The experimental observation of S3 band provides a better visualization of non-trivial topology of Bi(111)/Bi_2_Te_3_ films purely based on the ARPES measurements.

In summary, we studied the electronic structure of 30 nm Bi(111)/Bi_2_Te_3_ films. By observation of the third surface band near 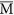 point, we found the directly experimental signature that Bi(111)/Bi_2_Te_3_ film can be topologically nontrivial. The origin of the nontrivial properties in Bi(111)/Bi_2_Te_3_ films needs further investigation in the future.

## Method

Bi_2_Te_3_ single crystals as well as Bi_2_Te_3_(111) films were used as the substrates. Bi_2_Te_3_ single crystals were cleaved *in situ* at 10 K. 40 nm Bi_2_Te_3_ (111) films were grown by MBE on Si(111) wafter. Bi(111) films grow as the biatomic layer (BL) growth mode. The thickness of each BL is about 0.39 nm. In order to get high quality films, we used a “two-step” growth method. Firstly, the substrate was kept at 250 K during the growth of the first 15 BLs^32^. Secondly, we raised substrate’s temperature to 420 K and grow more BLs. The deposition rate of Bi was about 0.3 BL/min. ARPES experiments were carried out in Advanced Light Source Beamline 12.0.1 at 10 K using a Scienta analyzer with the incident photons (h*ν*) of from 28 to 46 eV. The polycrystalline Au electronically contacted with sample was used as the reference of Fermi level. The energy resolution is about 10 meV and the angular resolution is better than 1% of the surface BZ.

## Additional Information

**How to cite this article**: Yao, M.-Y. *et al.* Topologically nontrivial bismuth(111) thin films. *Sci. Rep.* **6**, 21326; doi: 10.1038/srep21326 (2016).

## Figures and Tables

**Figure 1:**
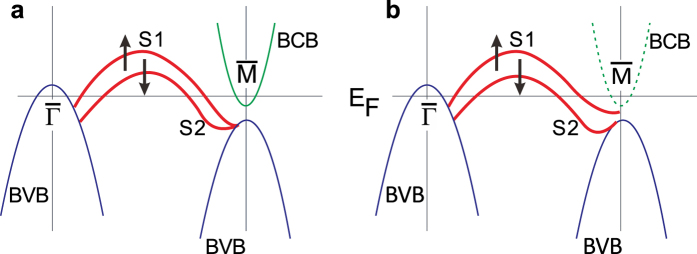
Sketch of energy bands of bulk Bi(111). (**a**) LDA calculations and (**b**) ARPES results. Red bands are spin splitting surface states, S1 and S2. Black arrows indicate the spin polarization. Blue and green bands are bulk valence band and bulk conduction band. Dashed line in (**b**) indicates that no bulk conduction band has been detected in ARPES. The agreement between LDA and ARPES near the 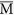 is poor.

**Figure 2:**
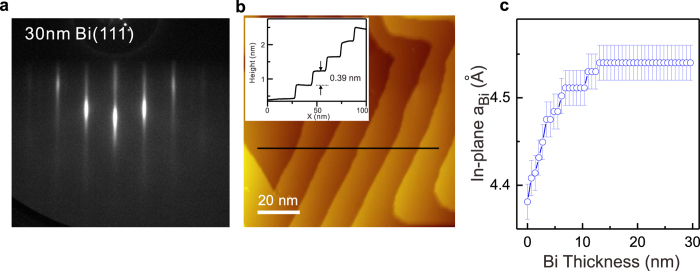
Characterization of Bi(111) film’s surface. (**a**) RHEED pattern and (**b**) STM topography of 30 nm Bi(111) films on Bi_2_Te_3_. Insert is the height line profile along the black curve. (**c**) In-plane lattice constant of Bi films as a function of the thickness. The high voltage of RHEED is 30kV. For the STM image, the bias voltage is 1.42 V, the tunneling current is 162 pA.

**Figure 3:**
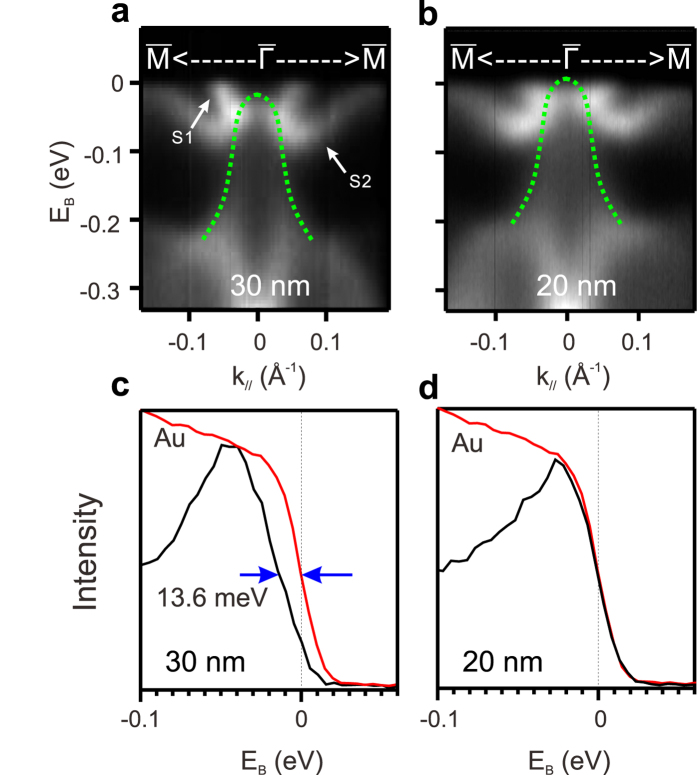
ARPES spectra near 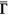 point. (**a**) ARPES spectra of 30 nm Bi(111)/Bi_2_Te_3_ film and (**b**) ARPES spectra of 20 nm Bi(111)/Bi_2_Te_3_ film along 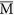
*-*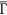-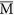 near 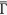 point. The incident photon energy h*ν* = 30 eV. Green dash lines mark bulk valence band. (**c**) Black and red lines are EDCs of 30nm Bi at 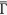 point and of Au. An energy gap was observed. (**d**) Black and and red lines are EDCs of 20 nm Bi at 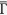 point and of Au. There is no energy gap.

**Figure 4:**
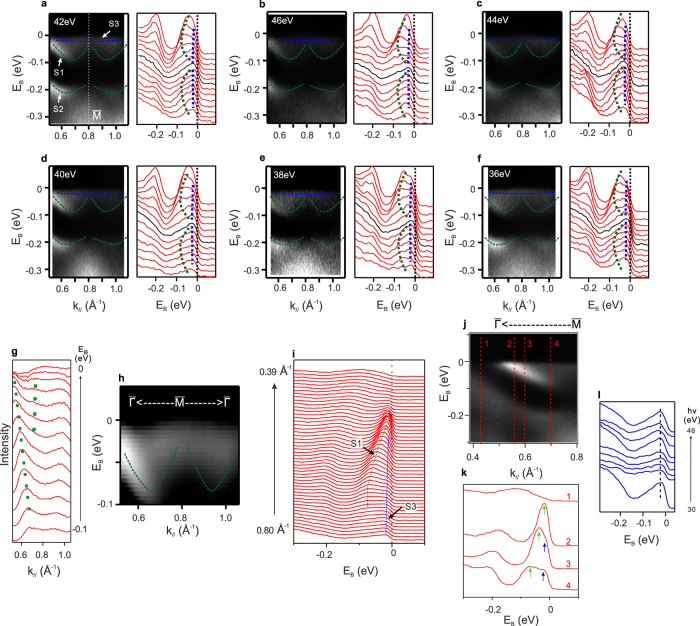
ARPES spectra near 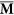 point. (**a**–**f**) ARPES spectra and corresponding EDCs of 30 nm Bi(111)/Bi_2_Te_3_ film near 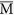 point along 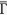-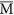-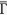 (incident photon energy hν = 42, 46, 44, 40, 38 and 36 eV). Green and blue dashed lines are the guides for the S1, S2 and S3 bands. (**g**) MDCs near Fermi level extracted from (**a**). Greed dotted lines mark MDC peaks of S1. (**h**) SDI plot of ARPES spectra (hν = 30 eV) near 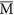 point along 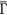-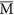-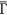 direction. Green dashed line shows the possible dispersion of S1 band. (**i**) High resolution EDCs along 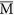-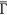 (hν = 30 eV). Green and blue dotted lines mark EDC peaks of S1 and S3 bands. (**j**) The corresponding ARPES spectra of (**i**). (**k**) Four EDCs from (**j**). The momentum positions are indicted by the red lines in (**j**) (k = 0.43 \AA^*−*1^, 0.56 \AA^*−*1^, 0.60 \AA^*−*1^, 0.70 \AA^*−*1^). (**l**) EDCs at 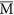 point as a function of the incident photon energy.

**Figure 5:**
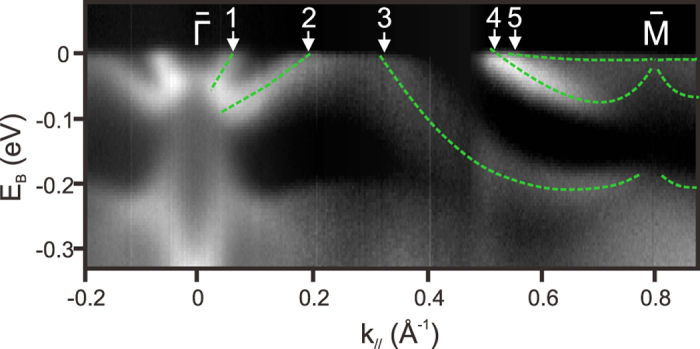
ARPES spectra from 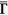 point to 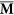 point. Green dashed lines mark the surface states. The white arrows mark the Fermi crossing positions of the surface bands. Odd number of crossings was observed.
